# Service robots in the mirror of reflective research

**DOI:** 10.1007/s10202-012-0111-8

**Published:** 2012-11-13

**Authors:** Michael Decker

**Affiliations:** Institute for Technology Assessment and Systems Analysis, Karlsruhe Institute of Technology, Karlstrasse 11, 76133 Karlsruhe, Germany

## Abstract

Service robotics has increasingly become the focus of reflective research on new technologies over the last decade. The current state of technology is characterized by prototypical robot systems developed for specific application scenarios outside factories. This has enabled context-based Science and Technology Studies and technology assessments of service robotic systems. This contribution describes the status quo of this reflective research as the starting point for interdisciplinary technology assessment (TA), taking account of TA studies and, in particular, of publications from the ethical and empirical social science perspective. Finally, based on this status quo, evaluation criteria for service robots are developed, which are relevant for further reflective research.

## Abstract (de)

Servicerobotik ist im letzten Jahrzehnt verstärkt in den Fokus der Reflexionsforschung zu neuen Technologien gerückt. Der Stand der Technik ist durch prototypische Robotersysteme geprägt, die für konkrete Anwendungsszenarien außerhalb von Fabrikhallen entwickelt wurden. Damit wurden kontextbezogene Science and Technology Studies und Technikfolgenbeurteilungen zu service-robotischen Systemen möglich. Dieser Beitrag beschreibt den Status Quo dieser Reflexionsforschung als Ausgangsbasis für eine interdisziplinäre Technikfolgenabschätzung (TA), wobei neben TA-Studien insbesondere Publikationen aus ethischer und empirisch-sozialwissenschaftlicher Perspektive berücksichtigt wurden. Abschließend werden auf der Basis dieses Status Quo Beurteilungskriterien für Serviceroboter entwickelt, die für eine weiterführende Reflexionsforschung relevant sind.

## Introduction

A multiperspective approach can be considered to be constituent for problem-oriented research in technology assessment (Grunwald 2010:19ff). The perspectives that are to be included, or rather the dimensions that could be used to judge the underlying situation, are for example (according to Bütschi et al. 2004) the (1) technical dimension, (2) political dimension, (3) societal dimension, (4) innovation dimension, and (5) knowledge dimension.

Regarding item 1, since service robotics is to be examined, the technical dimension is basically given. In the past few years, robotic research has developed systems that are capable of providing services,^1^ and it is the intended and unintended consequences of these technologies that are to be studied. An important precondition for doing this is to put service robotics in a context of use and to judge it in such contexts. The user contexts presented in this study have already been described in detail elsewhere (Decker et al. 2011). They are, for example, service robots in agriculture, service robots in telematics, and service robots in the care sector.

Regarding item 2, for the dimensions named above and at the level of generalization appropriate here, it can be said that service robotics can hardly be found on the current political agenda. If it is so, then at most as a topic of discussion in connection with medical care. However, service robotics has been a topic for research policy for a longer period of time, and various technology development programs on service robots (and on the related topic of ambient assisted living) and reflective programs on technology have already been conducted (see Sect. 2). The discussion of robots for military use plays a special role, leading to related issues of international policy (Singer 2009).

Regarding item 3, the societal dimension can be described as being relatively unagitated. Reports about service robots can be found frequently on the science pages of newspapers. The tenor of the reporting can be described as balanced to critical, or even as positive whenever concrete technological systems are presented.^2^ There are no major social controversies about service robots, and initial studies of their acceptance have been conducted (Sect. 2). For example, a museum robot that guides visitors meets with more interested curiosity than with surprise or anxiety. Science fiction literature and films appear to have prepared society for robots although robots are still a very infrequent manifestation in our life world (Christaller et al. 2001:28).

Regarding item 4, the degree of innovation demonstrated by service robot systems varies, with prototypes being one area of emphasis. Some of the systems are in an advanced laboratory state, which means that prototypes have been developed far enough that empirical research with probands is possible. The first commercially distributed prototypes are also on the market. Few service robot systems, such as vacuum cleaner robots, lawn mower robots, and even milking robots, can be considered as having reached the market (see Robo.org). Among these systems are also military applications such as the unmanned reconnaissance aircraft Global Hawk.

Regarding item 5, the last dimension, the knowledge available to us, is comparatively well documented from a technical perspective. The Robo Cup competitions (football, at home, and rescue), which can be interpreted as a benchmark, help us to reach an assessment. With regard to reflective research in general and to that for technology assessment in particular, the aim of this paper is to provide an overview of the status quo of available knowledge which in turn leads to the concluding discussion (Sect. 4).

## Technology assessment studies

An increasing amount of technology assessment research on robot systems has been undertaken in recent years. The Rathenau Institute, the TA-SWISS, and the Royal Academy^3^ are technology assessment institutions that have concerned themselves with service robotics in the recent past and continue to do so today.

The study “Robotics: Perspectives for Human Action in Future Society” (Christaller et al. 2001), published a decade ago, can be seen as signaling the resumption of technology assessment studies of robotics. Prior to that, especially in the 1970s and 1980s, three topics were central in connection with industrial robotics (see Christaller et al. 2001:14ff.):

- The change in work caused by industrial robots
- The economic aspects of industrial robotics
- The changes in the labor market caused by industrial robots.

As an example of the issues that were relevant in this period of time, in the following, I will cite from the study published by The Association of German Engineers (Verein Deutscher Ingenieure; VDI 1989) *Handlungsempfehlung: Sozialverträgliche Gestaltung von Automatisierungsvorhaben* (Recommendations for Action: Socially Acceptable Organization of Automatization Plans) because this study takes both the perspective of the worker and that of the entrepreneur into account. Among the principles that should be taken into account when developing robots are the following (VDI 1989):

- Early and continuous cooperation between planners, developers, and users
- Take corporate strategy into consideration
- Worker participation and participatory style of leadership
- Integration of those affected
- Early and comprehensive measures to provide information and training.

Christaller et al. (2001) have prepared a total of 16 recommendations for action regarding robotics in general. The recommendations for dealing with learning robot systems and the related issues of liability are of particular interest for service robotics. Matthias (2004) elaborated further on the issue of whether—and if so, to which degree—learning systems can lead to a responsibility gap. Christaller et al. (2001) suggested an insurance system for the use of robots that was oriented on ordinary liability insurance. The following recommendation was formulated with regard to liability itself (Christaller et al. 2001:219):

**Liability for robots** The owner of the robot is in principle liable for damage caused by the robot only if he or those assisting him are personally responsible. Such misconduct is present in particular if the organization, operation, and maintenance by the robot’s owner are faulty. The robot’s manufacturer is responsible for mistakes in the fabrication, construction, and instruction in the context of product liability.

The reversal in the burden of proof for faults and adequate demonstration of prima facie causality is supposed to make the robot’s owner co-responsible for the intransparency of the mechatronic system for third parties.

It is recommended that courts ease the assertion of claims for damages caused by robots within the framework of existing laws.

Another recommendation for action that is also relevant for service robots focuses on the control hierarchy in robot systems that work closely with humans and recommends that no care recipient is forced to accept the presence of a robot in his care environment against his will (“veto right”; Christaller et al. 2001: p. 220):

**Position of humans in the control hierarchy** The competence of persons to determine their own aims is fundamentally to be upheld in the context of robotics. The associated ban on human instrumentalization must be taken into consideration when establishing the respective decision-making hierarchy. The design of the man–machine interface and the program control are of great significance during the technical implementation of decision-making competence. To enable humans to take on responsibility for the functioning of robots, the robots must be controllable in the sense of being transparent, predictable, and responsive.

It is recommended that the respective persons are informed about all the cases in which a robot has its own sphere for decision making, and that the persons must provide their explicit or implicit approval. The refusal to provide such approval should have a veto function, especially for medical treatment and care.

In particular, this veto function has been challenged in various connections since it presumes the patient is cognitively fit (cf. the comments in Sect. 4 on dementia and autism). Individual recommendations have also been made for concrete fields of application, such as for the care sector relevant in this context (Christaller et al. 2001: p. 221):

**Robot assistants in care sectors** Examples for the use of robots in medical technology are computer-aided ventilators, novel aids for moving a patient to another bed, assistance systems for rehabilitation of human mobility, and assistant robots to facilitate the autonomous life of the aged and handicapped. When using robots in the care sectors, one must take into account that only humans should provide care in a responsible manner to other humans. Humans in need of care may not be treated as objects by removing human caregivers from their environment and replacing them by robots.

Our recommendation is, correspondingly, to employ robots only as tools or as technological assistants in providing care and maintaining the autarky of those needing care in their home environment.

A robot’s ability to act autonomously and also to learn from computer systems was analyzed in the TA-SWISS project “Die Verselbständigung des Computers” (The Computer Becomes Autonomous; Kündig and Bütschi 2008). This study focusses on computers as “embedded, networked, and autonomously acting computer systems” (Mattern and Langheinrich 2008). These autonomously acting information systems or even software agents can either take over services in the virtual world, such as search services or online trade, or they will become part of our everyday environment in the sense of ubiquitous computing or ambient assisted living. In the TA-SWISS study, this question receives a multidisciplinary answer. The liability for computer systems is also analyzed in depth in which autonomy is understood as the “lexibility that computers can adjust to changed circumstances without being reprogrammed by humans” (Rosenthal 2008:131). In that chapter, special reference is made to the obligation that due care be observed in programming systems since culpability—according to Rosenthal—is only present “if the liable party did not exercise the care that was objectively necessary given the circumstances” (Rosenthal 2008:133). The possibilities of liability without culpability are also dealt with (Rosenthal 2008:139ff.). Furthermore, issues of data privacy are covered in a separate chapter and discussed according to the issues of legality, transparency, proportionality, earmarking, integrity, and security (Baeriswyl 2008:120ff.). Electronic markets and nascent value-added chains are examined from an academic economic perspective. With regard to services, the Internet of services is examined in the development to an Internet of objects (Schmid 2008:107ff). And with regard to telematics in street traffic, the conclusion is “The transition from the old form of value-added systems to their new, Internet- and protocol-based form not only goes beyond the scope of a company but also that of a nation” (Schmid 2008:115). From the perspective of social science, Ingo Schulz-Schaeffer (2008:44) distinguishes three dimensions of autonomy: behavioral autonomy (the autonomous execution of behavioral programs), decision-making autonomy (the autonomous choice between alternative behaviors), and informational autonomy (here especially rule-generating self-control). Furthermore, he points out that there is a price to be paid for computers becoming autonomous. The result is autonomy of the means toward the ends, because the user also must put himself in the service of the machine. There is also a danger of technologically mediated heteronomy, because social constraints are frequently behind supposed technological constraints. This can result in a real lack of transparency, although in principle, control can be exercised over the system, as the close linkage and the complex interaction of the system components can lead to the generation of “behavior that is unpredictable overall” (Schulz-Schaeffer 2008:50).

In summary, the editors of the TA-SWISS study come to the conclusion that special attention should be paid to the following aspects of the increasing autonomy of technical systems (Kündig and Bütschi 2008:155):

- Consequences for the labor market
- Limits to rationalization, for example, in the care sectors, child raising, and education
- Limits to automatization because it can constrain the capacity for innovation
- Upheavals in the economy, for example, consequences for self-regulating mechanisms in the financial markets
- Consequences for the legal system if technological systems can make decisions autonomously.

Two European reports that can be considered relevant for technology assessment, particularly in the context of care, were also published in 2008. The IPTS (Institute for Prospective Technological Studies, Joint Research Centre, European Commission) published the study “Active Ageing and Independent Living Services: The Role of Information and Communication Technology” (Malanowski et al. 2008). It attributes a large potential for providing solutions to the information and communications technologies (5): “It is widely accepted that Information and Communication Technology (ICT) applications can provide new ways of helping older citizens to live independently.” In a comparison of need and technological offerings, the study points out that in the context of care, individual technological solutions have to be made available (Malanowski et al. 2008:25):

‘Design for All’ is a concept which consists of three strategies:

1. Products/services and applications should be usable by as many people as possible—regardless of age, ability or situation—without any modifications
2. Products should be easily adaptable to different users
3. Products should have standardized interfaces capable of being accessed by specialized users.

The concept links directly to the political concept of an inclusive society, which integrates all citizens into the information society.

Concrete policy options for being able to meet the social challenges posed by an aging society are developed in the IPTS study. One of the recommendations made there is an expansion of the empirical research in this field with special reference to users. It also advocates that the families of those affected (partners, children), care givers, and management are included, not just the aged who are affected.

The EU project “Robotics for Healthcare” (DG Information Society) includes the following items to the field of robotics for use in mental, cognitive, and social therapy (Butter et al. 2008:152):

- Monitoring systems
- Self-learning systems
- Game-based therapeutic systems
- Dementia-assistive systems
- Autonomous interactive systems.

These systems are thought to still be in the early stages of development, and even the advanced versions of these technological systems only suggest the potential that—according to these authors—is in these areas of application. This study suggests various topics for research that are on the interface between robotics and medical research and takes both social and economic developments into consideration. The technological aspects of (1) intuitive interaction with a robot, (2) sensor technologies, and (3) navigation control are identified as key areas of research for this type of robot. In summary, these authors reach relatively optimistic appraisals (Butter et al. 2008:156):

[…] designing systems that appeal to human emotions on exactly the right level require delicate design iterations. The success of the few preliminary systems illustrates that robotics have a large potential in this domain. […]. In the coming years, more knowledge will be gained on mental, cognitive, and social human interaction concepts. By implementing this knowledge in robot systems, these systems will act more and more like human beings and will be more and more able to support the development of skills. In this way, humans will be able to participate up to their potential in daily life.

The Royal Academy of Engineering conducted a study on autonomous technical systems in 2009, in which autonomously moving vehicles and artificial companions served as examples for ELSI studies, that is, those on ethical, legal, and social implications. For driver assistance systems, which the authors of the study see in a developing overall system from driver information systems via advanced driver assistance and cooperative vehicle highway systems to automated highways, the following issues are viewed as central from the perspective of technology assessment (Royal Academy 2009:7):

Will the development and uptake of autonomous vehicles marginalise road users in older vehicles? How can the autonomous highway be regulated? Who manages the road system, and how can autonomous vehicles from a variety of manufacturers and with individual owners be regulated? How will the insurance industry deal with responsibility for failures and accidents involving autonomous vehicles?

The experts, in their recommendations for acting on the second case, that is, on companions in the care sector, invite those involved to answer the following questions (Royal Academy 2009:11):

There should be engagement with older people on the use of technologies for allowing people to be monitored in their homes. Are they broadly welcomed, or is it likely they will lead to people feeling more abandoned, isolated and vulnerable than they currently do? What can be done to make these technologies work for users who might not routinely use computers? Are attitudes likely to change with the generations, so that people coming to retirement now and used to working in technology-driven environments may be more comfortable, so that by the time such technologies become mature they are also more accepted? Could an artificial companion ever be seen as offering real companionship—would it be any better or worse than a pet, for instance?

Finally, an ongoing TA-SWISS study should be mentioned here whose results will be presented in the course of 2012.^4^ It is also focused on applications in the care sector and is dedicated to the topic of “Robotics and Autonomous Devices in Social and Health Care.” It examines the opportunities and risks of using autonomous robots in the medical fields of care, rehabilitation, nursing, and therapy using the most realistic possible future scenarios. It refers to care provided to elderly people in a domestic environment or in institutions (homes, hospitals). The following research goals are mentioned:^5^

- Which applications exist already, for which are prototypes being tried out, and what conceivable fields of application are there for the future? What is the situation regarding the suitability of the devices in everyday life?
- Where is there a need for automation? Which actors and interest groups are behind the aim of delegating services previously performed by human beings to robots? To what extent do cultural backgrounds determine their acceptance in different areas?
- Is automation a practical solution? Is there a danger of jobs being cut in the social sector? Will robots supplant human beings who are not highly trained but often perform emotionally demanding tasks, for example, in nursing care?
- Is the advance of robots into the social sector acceptable from an ethical point of view? Or are there grounds for protecting a particular sector in which social interaction and emotions are important from mechanisation?
- How far should the pre-programmed autonomy of a robot be allowed to go without creating problems in terms of security? In this context the legal situation is important, and questions of liability should be discussed here.
- What economic potential is there, for example, for the manufacturers of such devices? And what about costs—in this respect, it is not only the purchase of the devices but also maintaining them that is important.
- Finally, the situation will be analyzed in an overall assessment. Based on this, recommendations will be formulated on how to deal with the problem, and this will be directed at decision makers, and in particular at politicians.

In addition to the technology assessment studies presented in this section, which are described here without any claim to completeness but with the goal of sketching the status quo, service robotics has also been at the focus of what is called the reflective sciences. Their findings are presented in following section.

## Reflective research on service robotics

Ethical reflection has increasingly concerned itself with service robotics in the last few years. At the end of 2006, the editors of the International Review of Information Ethics stated in the editorial to the special issue on “Ethics in Robotics” (Capurro et al. 2006): “Although robots are therefore progressively surrounding us in our professional lives as well as in our private sphere, we have only few reflections on the ethical and societal issues concerned with it.” In this special issue, Veruggio and Operto (2006) report on the origins of “roboethics” which took place at The First International Symposium on Roboethics, held in San Remo. Veruggio also served as the coordinator for the preparation of the “EURON Roboethics Roadmap” (Veruggio 2006), with which he wanted to trigger a debate to enable precautionary action (Veruggio and Operto 2006:7):

The aim of this roadmap is to open a debate on the ethical basis which should inspire the design and development of robots, to avoid to be forced to become conscious of the ethical basis under the pressure of grievous events. We believe that precaution should not produce paralysis of science and technology.

Some very fundamental questions, such as “What Should We Want From a Robot Ethic?” (Asaro 2006) and “When Is a Robot a Moral Agent?” (Sullins 2006),^6^ were raised during this phase of ethical reflection on robots in the same special issue.

In its final report (Capurro et al. 2008), the EU project “Ethicbots” referred to different cases (robot learning, military robots, social cognitive companions, surgery robotics, and a robotic cleaning system) and developed recommendations for action for each of them. It suggests a contextual handling of the responsibility gap, which should obviate regulation that is too strict. Comprehensive monitoring on the part of the EU is suggested for social companions, and it should be accompanied by reflective studies and technology assessment. The same applies to surgical robotics, for which it is suggested that technology assessment accompany development and that it also includes patients. The study deals furthermore with bionics and research on artificial intelligence.

The volume “*Robot Ethics. The Ethical and Social Implications of Robotics”* (Lin et al. 2012) was, according to a statement by the editor, the first coherent book on robot ethics to be published “that draws together such thinking on a wide range of issues such as programming design, military affairs, law, privacy, religion, health care, sex, psychology, robot rights and more.”^7^ “Medicine and Care” makes up a part of its own in this book. Borenstein and Pearson (2012) recommend that robots should not act alone in the care sector, even if their technical performance should clearly improve. Cooperation with nursing personnel is called a practicable route. Noel and Amanda Sharkey see limited possibilities for robot technology being used for providing care. While they do see an advantage for aged persons in assistant robots helping those needing care achieve greater autonomy and in companion robots, which for example help create opportunities for communication, helping to better maintain the social environment, they reject care robots for children (Sharkey and Sharkey 2012:279): “However, for children, although there may be benefits interacting with robots in social, educational, or therapeutic setting, robot childcare comes with too many risks to be considered viable.” Petersen (2012) finally distinguishes in his chapter, “Designing People to Serve,” five different cases that he discusses with reference, among other things, to the ban on human instrumentalization.^8^ In summary, the following aspects on the part of ethical reflection remain particularly relevant (Bekey 2012:20ff.):

- The fear of being replaced by a machine
- The dehumanization of work
- Current trends towards cooperative work
- Human interaction in healthcare, surgery and rehabilitation
- Robots as co-inhabitants; Humanoid robots
- Socially interactive robots
- Military robots.

Reflection on service robotics takes place not only in ethical reflection but also in the sociology of technology,^9^ which I will not discuss further here. Instead, I will present some initial empirical studies on service robots. A clear focus in empirical studies can be seen on applications in the care sector, although there are also individual studies on the acceptance of service robots in general.^10^ One current study on the acceptance of robots in the care sectors was published by Meyer (2011) under the title, *Mein Freund der Roboter. Servicerobotik für ältere Menschen*—*eine Antwort auf den demographischen Wandel?* (My friend the robot. Service robotics for older people. An answer to demographic change?). For the first time, this study reports on a comparative study of different application scenarios with different robot systems. It furthermore provides a comprehensive overview of the state of the art of the relevant technology (Meyer 2011:7ff.). In evaluating the status quo, the study reaches a positive result (Meyer 2011:133): “[…] has shown that service robotics does hold positive potentials for the aged: by facilitating their capacity to lead life autonomously, by supporting their health and safety at home, and—should nursing care become necessary—by decreasing their dependence and raising the quality of care.” In a quantitative analysis, the study comes to the result that applications of service robotics meet with the approval of more than half of the aged (Meyer 2011:137). This result is completely in line with the results of other studies (Friesdorf and Heine 2007; Meyer and Schulze 2009; Spellerberg et al. 2009). In the supplementary qualitative analysis, the author points out that in particular the acceptance of those robot systems that autonomously perform delimited activities in a household, such as vacuum cleaner robots and wiping robots, is particularly high (Meyer 2011:139):

The scenarios “health monitoring”, “fitness coach”, and “communication and stimulus” occupy medium spots in the acceptance ranking. After them come the “window cleaner robots”, the “therapeutic applications”, and “humanoid household robots for complex activities”.

The study points out that the technological autonomy of robots is an important factor for their acceptance. “Service robots are convincing for all the probands if they contribute to liberating those requiring care temporarily from their permanent dependence on human care providers (Meyer 2011:140). Finally, it emphasizes that technology can only support communication not replace it (Meyer 2011:141): “The interviews also show the deeply rooted anxiety the probands have about being confined with a robot and being left alone with it, ‘Pushed aside by one’s own relatives and forgotten by society, the final life span administered by man-machines’.”

In the project “Technology and Services in Demographic Change”, funded by the German Federal Ministry of Education and Research^11^ (Technologie und Dienstleistungen im demographischen Wandel, Bundesministerium für Bildung und Forschung, BMBF), user needs analyses were conducted, primarily on ambient assisted living (Bieber and Schwarz 2011). This project developed potential acceptance factors for “technological service innovations by senior citizens” in a qualitative manner (Hogreve et al. 2011).^12^ According to this, it is possible to distinguish three dimensions of acceptance, each of which with different acceptance factors (Hogreve et al. 2011:39).

- Technology-specific dimensions of acceptance (with the factors reliability of the technology, user friendliness, support by providers)
- Service-specific dimensions of acceptance (with the factors perceived utility, perceived quality, costs, perceived risk, confidence in vendor, trialability)
- User-specific dimensions of acceptance (with the factors technical affinity, sex, social environment).

The first of the five research topics in the ambient assisted living project for which the Ministry issued its call for proposals was for “fundamental issues of society with longer length of life” (BMBF 2011).^13^ Work on this topic is supposed to enlarge the knowledge base about demographic change, paying “special attention to the situation in which older people live, including the rapidly growing group of the very elderly” (BMBF 2011:5). It is also supposed to draft realistic conceptions of aging by conducting “research on the underlying cultural conditions for conceptions of aging and for the creation and spread of realistic conceptions of ageing” (BMBF 2011:5). Further topics in this field are recognition and defusing of generational conflicts and consideration of the acceptance of technological solutions. This 5-year funding program^14^ (until 2016) strives in general to achieve a comprehensive innovation-based approach (BMBF 2011:18): “Individual technological results are not at the forefront of funding, rather the implementation of innovative solutions that comprehend social, ethical, legal, and other social aspect and that are usually driven by user needs.” The recommendations of the expert group advising on this research program, as recorded in the Loccumer Memorandum,^15^ were thus implemented.

The WimiCare project is dedicated to the attempt to carry out technology development in a functional-participative manner in the care sector (Compagna et al. 2011,16). In this study, a driverless transportation vehicle and an assistant robot were employed in a stationary care institution for 14 days, and a transportation scenario and a drinks scenario were conducted with the residents, care personnel, and developers. In the transportation scenario, it was possible (Compagna et al. 2011:170) “on the one hand to achieve a significant reduction in the burden on the care personnel while on the other hand improving the overall situation in the care institution in the medium term.” The work of the assistant robot was also certified to be successful in the sense that, on the one hand, the patients were ready to interact with the robot, that is, to be addressed by the robot and to take a glass of water from it, if appropriate, and that “on the other hand, precisely residents with diagnosed (but not severe) senile dementia did not exhibit any haphephobia and interacted exceptionally well and in an uncomplicated manner with the artifact” (Compagna et al. 2011:172). In the conclusion of this study, the authors discuss the problematic of the methodological approach of introducing artifacts into the context of an application for just a short period of time (Compagna et al. 2011:173): “in interviews and discussions the residents of the institution never commented seriously about the possible use of new technologies in their institution.” It was possible to get the care personnel much better involved in coordination of the scenarios, and the robot developers, especially the marketers of the driverless transportation vehicle, were also able to draw benefit from the participatory development process.

The acceptance of a robot for measuring blood pressure was studied at the University of Auckland. The robot Charles is a natural language robot that explains to patients how they have to apply the blood pressure cuff that measures the blood pressure and tells them the result. Overall, nearly 60 people participated in the study. They were divided into two groups by age (those 45–65 years of age and those over 65) and independent of sex. The authors come to the conclusion that the differences in acceptance have less to do with age than with sex (Kuo et al. 2009:218):

This experiment investigated age and gender differences in people’s attitudes and reactions towards robots before and after interacting with the healthcare robot Charles. While the results showed that older people were less experienced with computers than the middle-aged, they had similar attitudes towards robots and rated the interaction similarly. There was a non-significant trend for older adults to be less comfortable during the blood pressure measurement. Men had significantly more positive attitudes towards healthcare.”

To obtain the different preferences with regard to health care robots, a survey was conducted among the residents of an old people’s home and the caregivers working in the home (Broadbent et al. 2009a, b). Of each group, 30 persons were questioned, however without there being a robot system present at the home. The questions referred to the appearance of the robot, and the respondents preferred a humanoid robot with arms, legs, and a head, a size of about 1.30 m, and silver color. There was no preference regarding male or female body shape or voice. Interesting are the differences in the two groups for the answers to questions regarding the care-giving tasks that a robot should take over (Broadbent et al. 2009a, b:647):

Residents prioritized healthcare tasks, e.g., making phone/video contacts to the doctor, reminders to take medications, helping people to get out of chairs, while staff prioritized assistance for their jobs, e.g., reminders for daily routine and drinking water, and escorting to meals.

An empirical study on the basis of focus groups confirms, according to its authors, these results (Hutson et al. 2011:584). In these focus groups, older people between the ages of 66 and 85 discuss different robot systems, which could be assigned to the categories machine type, animal type, and human type. Most of them were actually present, and only three were presented by video. The study gave priority to the psychological well-being of the users and contrasted this to functioning, which was considered necessary but precisely not sufficient (Hutson et al. 2011:579): “Among the different types of social robots, service type robots are designed to provide functional help; companion-type robots are designed to enhance psychological wellbeing.”

In the context of this study, information was collected from the domestic environment of several of the focus group participants. These participants were able to select one of the robot systems for use for ca. 7 days. An interview was conducted before and after this phase. The results of the focus groups and these home studies led to the formulation of demands regarding the function (e.g., natural language and establishing connections for communication with family members) and maintenance/servicing (e.g., battery capacity, washable, robust) of robot systems. The study came to the conclusion (Hutson et al. 2011:579):

We found that social robots have the potential to improve wellbeing in the elderly, but existing robots focus more on healthcare and healthy behaviour among the elderly. Based on our focus groups and home studies we produced a set of requirements for social robots that reduce loneliness and improve psychological wellbeing among elderly.

Sparrow and Sparrow (2006:156) express themselves critically to such findings obtained in laboratory situations in their paper “In the hands of machines? The future of aged care.”

We also believe that there is likely to be a big difference between laboratory tests and commercial use of robots in this context, with the conditions that would need to be met for the real-world application of robots, in terms of the robustness, reliability and cost of robot carers, being much more demanding than laboratory tests reveal.

In addition to the arguments already mentioned that the introduction of service robots can be expected to be accompanied by a reduction in human care providers and the fear that a patient’s or user’s autonomy will rather be limited instead of expanded, they point out that demographic change can be described differently (cf. Section 2):

Too often, in our society, older persons are considered only as problems or as objects of study, rather than as full citizens with a valuable contribution to make to the community. The desires and opinions of older people themselves are neglected in favour of the expertise of gerontologists, sociologists and economists; the deeper philosophical questions concerning the meaning of the end of life experience are passed over in favour of concentrating on achieving technical solutions to problems defined in terms amenable to such solutions.

This list of reflective studies on service robots in general and on their application in the care sector in particular shows that service robots are already in the focus of reflective research. A number of analyses have already been conducted and also various recommendations for action developed. The discussion of the opportunities and risks associated with service robots has begun, with most studies reaching the conclusion that context-based individual studies are needed to be able to study the intended and unintended consequences.^17^ The studies also point out that the reliability of the machines, the concrete implementation of the usage and feeling comfortable in using the technology are central aspects—as are the economic and legal feasibility—that must be analyzed. In the concluding discussion, the individual points that were identified in the technology assessment studies and in the general discussion of ethics and social science will be summarized.

## Discussion

The results of both the technology assessment studies and reflective research distinguish between the relevant aspects of robotics, such as the external and inner appearance of robot systems, for example, humanoid nature and capacity to speak. I will discuss this in the following. The robot’s external appearance is considered decisive for the expectations that a human user has when encountering it. Expectations are relatively high, if the robot has a high-tech appearance and can, in contrast, be kept lower than if it has a childlike appearance (Broadbent et al. 2009b). Just, the opposite may be true of a user’s confidence in a robot. The authors assume that this could be one of the reasons why a toylike robot was not able to attract much interest among users in a hospital. Coeckelbergh (2009) recommends putting external appearance at the forefront of an ethical appraisal of service robots since—according to him—it is decisive how they work on people and which consequences result from this for human-robot interaction. Salvini et al. (2010) point out that appearance exerts a significant influence on perceived utility. This aspect can be taken into consideration in the development of service robots in order to increase their acceptance. In doing so, the goal must be to reduce the feelings of discomfort of those directly or indirectly involved. The team of authors recommends that above all four aspects have to be taken into consideration. First, the robot’s external appearance should correspond to its function and task. It should also radiate friendliness in order to arouse positive emotions. Furthermore, an attractive and pleasant appearance is advantageous, just as is perceived safety, which triggers a soothing feeling among participants.

The size of a robot plays a role with regard to individual aspects of its external appearance. At first the issue is naturally the technically necessary size needed by the robot to perform its task. At the same time, several studies confirm that smaller robots are viewed as advantageous (Giulini et al. 2005; Broadbent et al. 2009b:324; Meyer 2011:139), which can be interpreted overall as “as small as possible.”

The humanoid shape of robots is generally not viewed as being helpful. The majority of the probands preferred robots that did not look human (Arras and Cerqui 2005; Oestreicher 2007), yet here too, the context of the application should be taken into consideration. Also, robots without a face are preferred. That a humanoid appearance can make a difference can be deduced from studies of the sex of humanoid robots, in which differences in user behavior were noted with regard to android and gynoid robots (Powers et al. 2005).

Voice and the capacity for natural language should serve here as the transition from a robot’s external appearance to its inner appearance, since—depending on how the border is drawn—they are counted on the one hand as belonging to the external and on the other to the internal qualities. Communicative capacity is considered decisive for how the user rates the robot. The knowledge and “social position” of a communicative robot that can speak are valued higher. This also has an influence on whether users accept or follow, for example, pointers or requests from the robot. In general, both male and female users accept a robot whose “personality” (“extrovertedness”) is similar to their own (Broadbent et al. 2009b:324). The personality or the demeanor of a robot is supposed to be adjusted to the task that the robot should perform. In an empirical study in which a robot could be equipped with two different personalities—one funny and one serious—the robot was supposed to stimulate the probands to exercise. The probands said that while they liked the funny robot better, they performed their exercises better with the serious robot (Goetz and Kiesler 2002). Hendriks et al. (2011) point out in a rather general way that users of vacuum cleaner robots desire a calm one that gives the impression of having the situation under control. It should demonstrate cooperative behavior, act according to routines, and be systematic. The authors conclude from this that users thus most of all desire a robot that performs its work, not a particularly unusual device.

Many studies point not only to the internal and external appearance but also to the necessity of a technology assessment discussion of the topic of service robotics.^18^ In particular, they bank on users being included, in the sense of a technology assessment that accompanies technological developments. Hendriks et al. (2011) recommend first determining the wishes of the target group with regard to the demeanor of a service robot, translating this into behavior, and adjusting this until the desired result is reached by having it evaluated multiple times by potential users during the developmental phase. The way robots are judged by users depends strongly on the user’s needs. If users need help or if the robots promote their autonomy, then, the robots will be judged more positively. What is decisive—and on this, the studies generally agree— the capacity to adapt, that is, the capacity of the robot system to learn. This capacity is important both in a first phase of adjustment (initial installation) to an individual user and in the constant adjustment to a changing user (Broadbent et al. 2009a, b), while explicitly appealing to professional users, such as in the care sector (Ceccarelli 2011; Compagna et al. 2011).

The question as to the legal framework within which service robots are supposed to act is raised very frequently and almost always in connection with the capacity for adapting. Sharkey and Sharkey (2012), for instance, demand that ethical reflection take place and that the legal foundations for the use of service robots be developed. According to their view, it is unclear who is responsible for the actions of the robot, in the case of an accident or malfunctions. Salvini et al. (2010) just like the technology assessment studies mentioned above, points out that while industrial robots are well covered from a legal point of view, robots on the street, for instance, are simply not taken into consideration. They refer to the Vienna agreement on street traffic, stating, for example, that every mobile object in street traffic needs a driver. Robots have to be defined as new participants in traffic, and new rules have to be created. Furthermore, regulations are needed for the sensitive area of data privacy, the private sphere, and monitoring. These results from the fact that in the future service robots will be able to move about in public space equipped with cameras. Clear guidelines are also lacking for this.

This is a reference to the data privacy aspects that were addressed in connection with technology assessment. And precisely in the care sector, according to Sharkey and Sharkey, a very close analysis is needed regarding which data can be seen from whom and when. The possibilities of physiological computing (or biocybernetic adaptation) create technological opportunities that emphasize the urgency of such regulation to protect the private sphere (Böhle et al. 2012a, b). While the most empirical studies have been conducted precisely in this field (see Sect. 2), their results have not yet been scrutinized and their plausibility must still be examined. Most studies thus point out that it is considered desirable for older people to remain in their familiar social environment as long as possible, and that technological aids could support precisely this factor. On the other hand, studies (Carstensen et al. 2003; Charles and Carstensen 2010) indicate that aging can alternatively be understood as a process of adjustment in which a stronger and stronger selection of social contacts—so to speak down to the essentials—can be observed. Furthermore, first positive findings in therapeutic research on people suffering from dementia (Compagna et al. 2011) and on autistic children (Robins et al. 2009) indicate that application potential might also be found here, even though various studies consider that precisely the immediate work with people must be carried out by people.

In conclusion, let me mention that the consequences for the labor market and for work processes are relevant issues that will have to be studied in connection with service robots. Ultimately service robots carry out, at least in part, activities that previously have been performed by humans. For these actions human workers will be replaced which can have relevant consequences on the labor market. In any case, the factor can lead to rejection among employees and also political decision makers (Salvini et al. 2010). The references above to the inclusion of professional users (e.g., care providers) in the development of technology indicate a change in work processes that could be linked to service robots. Precisely the lifting and bending by care providers have been identified as being particularly relevant, and they are considered of special importance in connection with decisions about leaving the profession (Hasselhorn et al. 2005). In this respect, the issue is to consider the entire context of action instead of the singular replacement of an activity, and to make recommendations in the context of an optimization of the entire situation (Böhle et al. 2012b).

Sung et al. (2010), in their empirical study of robots, examined the domestic environment as the place of work of service robots. They were able to identify five patterns of interaction between humans and robots in this context. First, the robot is a tool that has to fulfill a purpose. Second, as a result, it is an agent that exerts a direct influence on the domestic environment. It, furthermore, leads humans to change their environment. In addition, it can promote social relations and lead people in its environment to undertake social activities since under circumstances these people attribute capacities to it that are similar to those of a human. Alone these patterns of interaction indicate that only a detailed context-related analysis of the use of service robots can provide information about the conditions of innovation and side effects. The diverseness of the opportunities for using service robots in combination with the numerous potential users constitutes a special challenge to technology assessment.

## Footnotes

1 This is thus an approach to technology assessment from the perspective of push technology.

2 For example: http://www.spiegel.de/wissenschaft/technik/roboter-obelix-erkundet-freiburg-a-851322.html (October 2012).

3 The Royal Academy would presumably not view itself as a technology assessment institution, yet the report cited here can be considered a technology assessment study, especially since concrete recommendations for action are also provided.

4 The Dutch Rathenau Institute is also supposed to present the results of the project “Robots everywhere” in 2012, which also deals with social or service robotics. See http://www.rathenau.nl/en/themes/theme/project/robots-everywhere.html (29 October 2012).

5 http://www.ta-swiss.ch/projekte/biotechnologie-und-medizin/robotik/ and http://www.ta-swiss.ch/en/projects/biotechnology-medicine/robotics/(found 4.2.2012).

6 According to Nakada (2012) the relevance of roboethics for Japanese students of ethics of the information society is questionable, in contrast (p. 159).

7 The fact that robotics is currently considered an interesting topic for ethical reflection can be documented here by the fact that this “first volume” is immediately being followed by another (Decker and Gutmann 2012).

8 cf. Decker (2008).

9 As an example, let me mention here Rammert and Schulz-Schaeffer (2002) and Weyer (2006) and refer the reader to the references given there and to the current Focus article of the TATuP-journal on: *Parasoziale Beziehungen mit pseudointelligenten Softwareagenten und Robotern. Intelligente Artefakte als Herausforderung für Soziologie und TA* including seven contributions (Parasocial Relations with Pseudointelligent Software Agents and Robots: Intelligent Artifacts as a Challenge to Sociology and TA; Böhle and Pfadenhauer 2011).

10 For example: Salvini et al. (2010), Sung et al. (2010), Hendriks et al. (2011).

11 Research on information and communication technology is also established at the European level. As early as 1988, the two HELIOS research programmes “for disabled people” were started and this research has been continued since then with a focus on the field of ambient assisted living.

12 Other projects in this area of priority funding examine, for example, the “user acceptance of assistance systems” (Lutherdt et al. 2011) and of “the aging of residences together with the resident” (Viehweger et al. 2011).

13 The other topics are: utilizing the competence and experience of the elderly for the economy and society, aging in good health, social participation: staying mobile and in contact, living safely and autonomously.

14 An overview of the technical projects can be seen in the Federal Ministry of Education and Research (BMBF 2012).

15 Loccumer Memorandum: Technische Assistenzsysteme für den demographischen Wandel—eine generationenübergreifende Innovationsstrategie (Technical Assistance Systems for Demographic Change: A Multigeneration Strategy for Innovation). AAL Expert Council of the BMBF, 2010.

16 See also http://www.wimi-care.de (October 2012).

17 For example, Böhle et al. (2012a, b) suggest considering care arrangements as such contexts.

18 For example, Coeckelbergh (2009), Sung et al. (2010), Salvini et al. (2010) and Ceccarelli (2011).
